# Sediment deposition within cascade reservoirs: a case study of Baihetan Reservoir in the lower Jinshajiang River, China

**DOI:** 10.1038/s41598-023-48052-1

**Published:** 2023-11-24

**Authors:** Jun Li, Yulei Gao, Lei Cao, Xianyong Dong, Yaochang Ma, Yahui Zheng

**Affiliations:** 1 Upper Changjiang River Bureau of Hydrological and Water Resources Survey, Chongqing, 400021 China; 2 River Basin Complex Administration Center, China Three Gorges Corporation, Yichang, 443113 China; 3 China Three Gorges Construction Engineering Corporation, Chengdu, 610095 China; 4 Bureau of Hydrology, Changjiang Water Resources Commission, Wuhan, 430010 China

**Keywords:** Hydrology, Solid Earth sciences

## Abstract

Sediment deposition in cascade reservoirs is not only related to the utilization efficiency of the reservoir itself but also to the boundary conditions for the operation of other reservoirs in the same group. The Baihetan Reservoir is the largest hydropower project with the highest unit capacity in the world, and it is necessary to consider sediment deposition within it, as this affects the comprehensive operation of cascade reservoirs in the lower Jinshajiang River. In this study, the input water, sediment, and deposition characteristics were analyzed based on both field hydrological and topographic data of the Baihetan Reservoir during its initial impoundment period. The results showed that water entering Baihetan Reservoir was mainly derived from the upper main stream, and approximately 41% was concentrated in the third quarter. Ten times the amount of sediment derived from the main stream was received from tributaries and uncontrolled areas of the reservoir, and these are the main sediment input sources. The fluctuating backwater area influenced by the upstream Wudongde Reservoir was slightly eroded, and siltation mainly occurred in the dead storage capacity (below 765 m) of the main stream and tributary estuaries in the perennial backwater area; approximately 15.8 times that in the regulating storage capacity (between 765 and 785 m). The differences between the results of this study and those from the reservoir demonstration stage indicate that was a lack of understanding about how climate change, human activities, and uncontrolled areas would affect siltation patterns. In future projects, research focusing on climate trend analyses and the comprehensive consideration of human activities should be combined with extensive sediment production monitoring and model parameter calibration.

## Introduction

With the increasing demand for clean energy, cascade reservoirs have become an important means of developing and utilizing water energy resources in rivers. However, the operation of cascade reservoirs in a watershed not only significantly impacts the reservoir area itself, but it also has a series of impacts on the water and sediment conditions^1^, river evolution^2,3^, flood control^4,5^, water supply^6,7^, ecology^8^, power generation^9^, and shipping^10–12^ of both the reservoir group and the downstream rivers. Therefore, the operation of cascade reservoirs has shifted from focusing on optimizing operations on a single spatiotemporal scale to the multi-objective comprehensive consideration of factors such as flood protection, power supplies, water resources, the aquatic ecology, and shipping on variable spatiotemporal scales^13–16^. However, these factors are also affected by various impacts and risks associated with hydrological and meteorological factors^17,18^, scheduling modes^19,20^, supply–demand contradictions^21^, and power grid topology^22,23^. In addition, it is difficult to estimate and understand sediment deposition when designing and managing reservoirs, and deposition not only reduces the utilization efficiency of the reservoir but it has also led to reservoir failure and abandonment. The joint operation of cascade reservoirs not only affects the utilization efficiency of a specific reservoir, but it also affects the sediment boundaries of other reservoirs in the group^24–26^, affects the comprehensive benefits of the entire cascade reservoir system, and can affect the regional water ecological environment^27–29^. Sediment deposition is thus a key factor to be considered when designing and monitoring the operation of multi-reservoirs^30^, and in-depth research is required to ensure that cascade reservoir benefits are optimized.

Six cascade reservoirs are operational on the upper Yangtze River (UYR), which is the world’s largest hydroelectric river, and a total annual power amount of 2.63 × 10^11^ kWh is generated, which makes the Yangtze River Basin (YRB) the largest source of clean energy in the world^31^. In the joint dispatch and management of controlled water projects in the YRB approved by the Ministry of Water Resources of China in 2023^32^, 125 water projects within the main tributaries of the Yangtze River will be included in the scope of joint dispatch. Hydropower development in the UYR has increased since the Three Gorges Reservoir (TGR) became operational in 2003; a super-large-scale reservoir group will now be built, and its sediment retention effect will sharply change the sediment conditions of the TGR. As sediment deposition has become an extremely important constraining factor for large reservoir groups, sediment deposition in the YRB (particularly in the cascade reservoirs of the Jinshajiang River) has become a considerable research focus, and many studies have been conducted.

In this respect, the Brune sediment retention rate grouping method was used to analyze the sediment retention effect of 66 reservoirs involved in the cascade reservoir group in the UYR^33^, and Li et al.^34^ investigated the rate and distribution of sedimentation in the TGR. With advancements in the cascade reservoir construction process, balancing the dynamic use of storing clean water and discharging muddy water during the flood period has been ascertained using typical measurements of water and sediment processes^35,36^, and the characteristics of flow and sediment changes, as well as the sediment detention of the cascade reservoirs in the LJR have also been analyzed^37,38^. In addition, prediction research on the sedimentation balance of the TGR under new water and sedimentation conditions has been conducted^39^. Furthermore, following the operation of the Xiangjiaba and Xiluodu Reservoirs in the LJR, the sedimentation of the two reservoirs and the law of channel evolution downstream the dam have been analyzed^40,41^, and Gao et al.^42^ analyzed the flow and sediment properties inputted into the TGR and changes in the source composition, focusing on the high flood period influenced by cascade reservoirs in the LJR. The Baihetan Reservoir was constructed as part of the second stage of building four cascade power stations in the LJR; it has the second largest installed capacity in the world, and sedimentation is significant. In the design stage, Lin et al.^43^ established a one-dimensional numerical model to calculate sedimentation in different operational periods following its completion, and Yin et al.^44^ conducted a model test study on the bed load sediment at the outlet of cascade reservoirs in the LJR.

However, studies on sedimentation in the Baihetan Reservoir were mostly based on the use of simulation predictions during the design stage, whereas relatively minimal research on actual sedimentation has been conducted. The Baihetan Reservoir has operational for more than two years since the initial water was officially stored on April 6, 2021, and many hydrological and topographic observations have been conducted during this period. As the largest sediment yield intensities of the Baihetan Reservoir area occur in sections and tributaries of the LJR, the sediment deposition characteristics of these areas are thus representative. Therefore, rich hydrological and topographic observation data from the Baihetan Reservoir area were used in this study to analyze the characteristics of sediment deposition during the initial period since its inception. The results are expected to be useful for the optimum operation of the Baihetan Reservoir and other cascade hydropower stations in the basin.

## Materials and methods

### Study area

**Figure 1:**
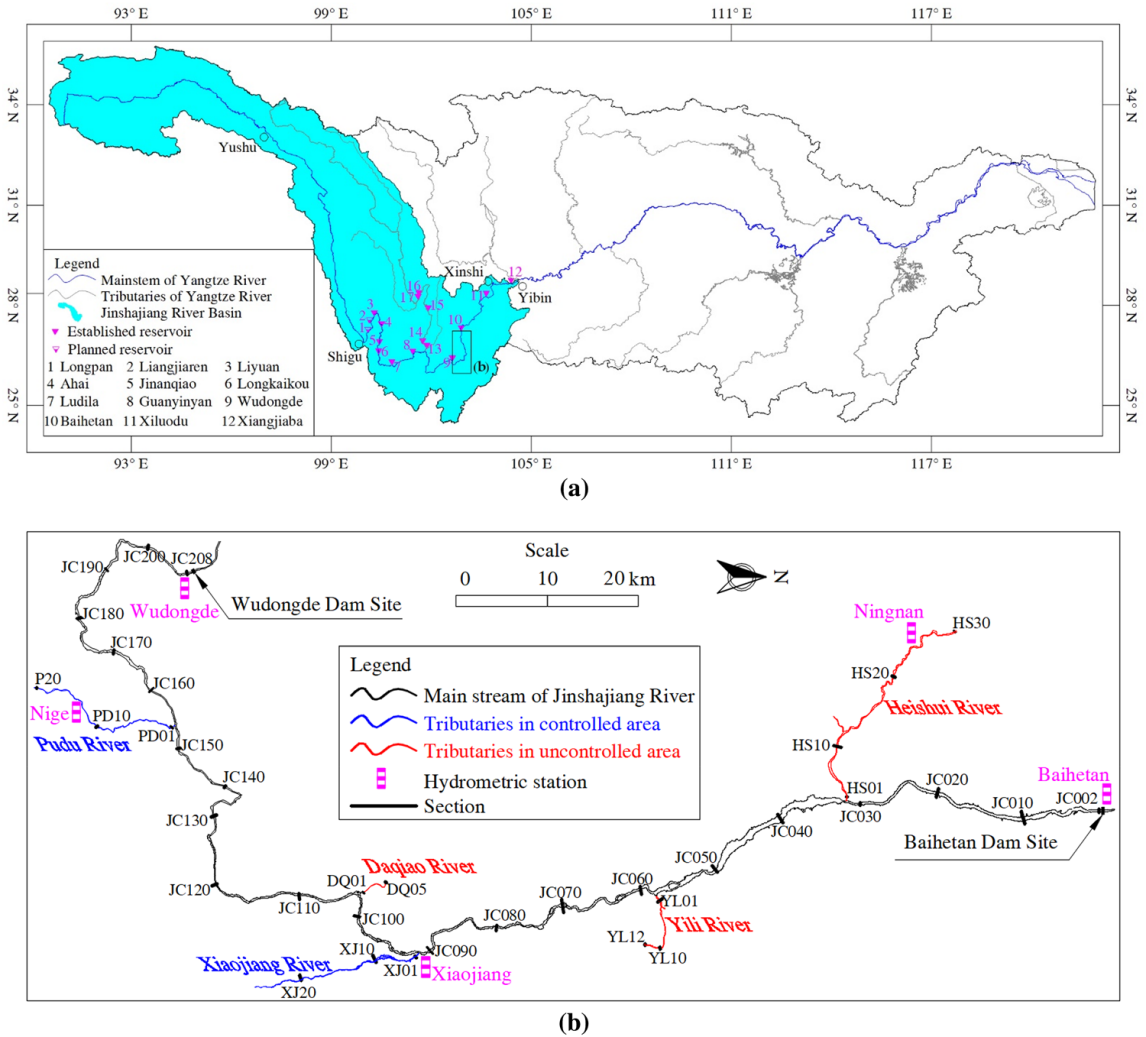
Study area: (**a**) drainage areas of the Yangtze River; (**b**) river system of Baihetan Reservoir.

Baihetan Reservoir (as shown in Fig. 1) is the second stage of the four cascade reservoirs in the LJR. It is situated approximately 180 km from the upper Wudongde Reservoir and 200 km from the lower Xiluodu Dam. It has a hydroelectric capacity of 16 million kW and an average annual generating capacity of 6.24 × 10^10^ kW. Initial water storage with a level of 727.07 m officially began on April 6, 2021, and the level at the front of the dam was then increased to 816.61 m on September 30, 2021. Water storage began at midnight on August 1, 2022, with an initial level of 775.63 m. The highest water level reached 825.00 m on October 24, at 18:00, and the reservoir has a cumulative storage capacity of 8.9 × 10^9^ m^3^.

The first-level tributaries with lengths of more than 500 km^2^ on the left and right banks of the Baihetan Reservoir area include the Pudu, Xiaojiang, and Yili rivers (right bank) and the Daqiao and Heishui Rivers (left bank) (Fig. 1b, Table 1). The basin controlling Baihetan Reservoir has an area of 4.3 × 10^5^ km^2^, and the basin area associated with its interval has an area of 2.42 × 10^4^ km^2^. Except for the five major tributaries, the total confluence area of small- and medium-sized tributaries is approximately 3 × 10^3^ km^2^.

**Table 1:** Location and measurements of first level tributaries in the Baihetan Reservoir area.

Name	Bank side	Length (km)	Basin area (km^2^)	Natural bed drop (m)	Reflective hydrometric station
Pudu river	Right	380	11,090	1850	Nige
Daqiao river	Left	70	802	2480	–
Xiaojiang river	Right	134	3120	1510	Xiaojiang
Yili river	Right	121	2560	2110	–
Heishui river	Left	174	3600	2460	Ningnan*

*Observations at Ningnan were halted in 2021 because of the influence of reservoir backwater.

### Data and analysis

The data adopted in this study include fixed cross-sectional terrain data and flow and sediment data measured in Baihetan Reservoir since storage began in 2021.

A total of 207 sections, with an average section spacing of approximately 830 m, were observed in the trunk stream of the reservoir. There were 11, 3, 23, 13, and 34 sections in the tributaries of the Pudu, Daqiao, Xiaojiang, Yili, and Heishui Rivers, respectively, and the average section spacing was approximately 750 m.

Wudongde and Baihetan stations record the inflow and outflow of the main Jinshajiang River in the Baihetan Reservoir area, as shown in Fig. 1b, while Nige (established in 2009), Xiaojiang (established in 2020), and Ningnan stations (established in 1970) record the inflow and outflow of the Pudu, Xiaojiang, and Heishui Rivers, respectively. The water and sediment data include the two-year daily average flow discharge and suspended-sediment concentration (SSC) data from five hydrometric stations in the main river and its tributaries. The annual values from Xiaojiang station in 2020 and the multi-year average values from Nige station from 2010 to 2021 were also collected. Owing to inundation of the Baihetan Reservoir, testing was halted at the Ningnan hydrometric station on the Heishui River by 2021. Therefore, water and sediment values of Heishui River were based on multi-year averaged values obtained from 2011 to 2020. As there are no hydrometric stations to describe the runoff and sediment, the Daqiao River basin, the Yili River basin, and the Heishui River basin are referred to as “uncontrolled areas” in this study.

During sediment deposition analysis, the amount of sedimentation was calculated using the volume method^45^ based on cross-sectional terrain data.

## Results

### Runoff and sediment load

#### Main stream

**Figure 2:**
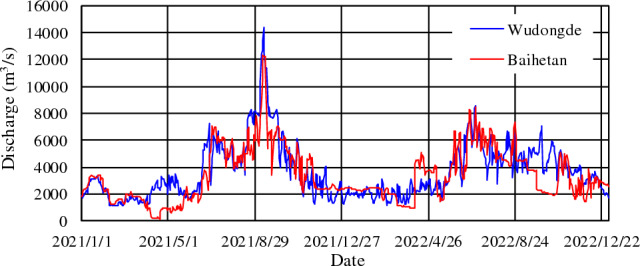
Daily averaged inflow and outflow discharge process of the mainstream in the Baihetan Reservoir area.

**Figure 3:**
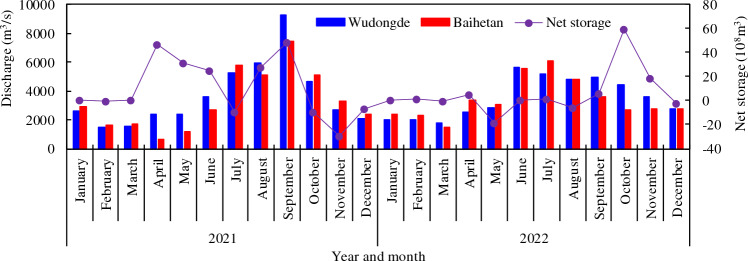
Monthly averaged inflow and outflow discharge from Baihetan Reservoir and net storage.

In 2021, the maximum inflow and outflow of Baihetan reservoir occurred in mid-September and October, respectively (Table 2, Fig. 2). The maximum inflow of 16,100 m^3^/s (September 10) was recorded at Wudongde hydrometric station, and the maximum outflow of 13,700 m^3^/s (October 10) was recorded at Baihetan hydrometric station. The inflow peaked in mid-September. Due to water being stored within Baihetan Reservoir, the annual average flow rate decreased from 3680 m^3^/s at Wudongde to 3340 m^3^/s at Baihetan. The annual intake and output runoff were 1161 × 10^8^ m^3^ and 1055 × 10^8^ m^3^, respectively. The storage process at Baihetan Reservoir was balanced from January to March before the flood period, and the water input and output was relatively equal. The main reservoir storage period was from April to September (excluding July), with a total storage runoff of approximately 179.88 × 10^8^ m^3^. The maximum storage capacity was 48.64 × 10^8^ m^3^ in September, followed by that in April. From October to December after the flood, the outflow discharged from the reservoir increased by approximately 45.72 × 10^8^ m^3^. Approximately 124.71 × 10^8^ m^3^ of water was retained by the reservoir throughout the year. Correspondingly, the monthly average flow discharge at Baihetan in April, May, June, August, and September was lower than that at Wudongde, from 800 to 1799 m^3^/s, but it was 310–590 m^3^/s higher than that at Wudongde from October to December (as shown in Fig. 3).

**Table 2:** Characteristic discharge values from the Baihetan Reservoir area.

Station	Year	Annual discharge (m^3^/s)	Runoff (10^8^m^3^)		Maximum discharge		Minimum discharge	
			Annual	July to September	Value (m^3^/s)	Occurrence	Value (m^3^/s)	Value (m^3^/s)
Wudongde	2021	3680	1161	540	16,100	September 10	596	June 3
	2022	3560	1122	397	9210	October 25	867	October 29
Baihetan	2021	3340	1055	487	13,700	October 10	2.84	May 6
	2022	3400	1071	385	13,700	October 31	602	May 22

**Figure 4:**
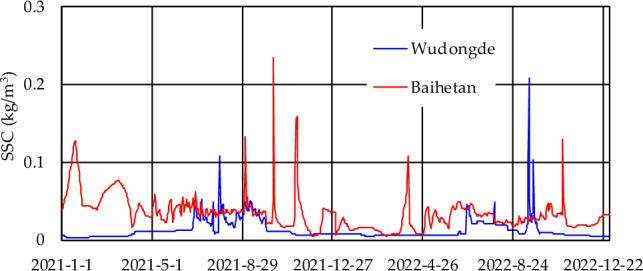
Intake and output processes of mainstream SSCs in the Baihetan Reservoir area.

The statistical characteristics of the SSC and associated process are presented in Table 3 and Fig. 4. Despite the influence of the inflow of tributaries and the operation of Wudongde Reservoir, the amount of sediment discharged from Wudongde was still lower than that from Baihetan, with annual sediment discharges of 226 × 10^4^ t and 438 × 10^4^ t, respectively. The sand peak discharged from Wudongde occurred on July 31, with a maximum SSC of 0.524 kg/m^3^, and a maximum SSC amount of 0.054 kg/m^3^ was discharged during the September flood period. The sand peak discharged from Baihetan occurred on October 10, with a maximum SSC of 0.743 kg/m^3^. During the main impoundment period of the reservoir, a maximum SSC output of 0.373 kg/m^3^ occurred on September 3. The minimum annual SSC discharged from Wudongde and Baihetan occurred during the dry season.

**Table 3:** Characteristic discharge values within the Baihetan Reservoir area.

Station	Year	Annual concentration (kg/m^3^)	Amount (10^4^ t)		Maximum concentration		Minimum concentration	
			Annual	July to September	Value (kg/m^3^)	Occurrence	Value (kg/m^3^)	Value (m^3^/s)
Wudongde	2021	0.020	226	170	0.524	July 31	0.002	January 9
	2022	0.013	146	89	0.515	September 21	0.004	February 9
Baihetan	2021	0.042	438	185	0.743	October 10	0.004	December 1
	2022	0.026	283	106	0.607	October 30	0.004	March 8

In 2022, the maximum inflow 9210 m^3^/s and outflow 13,700 m^3^/s from and to Baihetan Reservoir occurred on October 25 and 31, respectively (Table 2, Fig. 2). The outflow from September to November after the flood season was significantly lower than the inflow. Similar to 2021, no significant storage process occurred before April. From May to the end of July, the reservoir operating water level was in a period of decline, decreasing from 800 to 775 m. From August 1st, the reservoir began to fill, with the main storage periods concentrated in September, October, and November, when the total intercepted runoff was approximately 89 × 10^8^ m^3^. Accordingly, the average monthly flow at Baihetan in January, February, April, May, and July was larger than that at Wudongde, ranging from 180 to 880 m^3^/s, whereas that from September to November was smaller, ranging from 850 to 1770 m^3^/s (Fig. 3).

In 2022, the amount of sediment discharge inputting the reservoir at Wudongde station was still lower than that of outputting at Baihetan (Table 3, Fig. 4), input of 146 × 10^4^ t and output of 283 × 10^4^ t, respectively. The sand peak with a maximum SSC of 0.607 kg/m^3^ was recorded at Wudongde station on October 30, which was synchronous with the maximum flow process. The peak SSC was 0.366 kg/m^3^, and it corresponded with the peak flood occurring at the end of June. The sand peak with a maximum SSC of 0.515 kg/m^3^ was recorded at Baihetan on September 21. The peak SSC at the end of June was 0.049 kg/m^3^, and the minimum SSC was recorded at Wudongde and Baihetan during the dry season.

An analysis of the monthly distribution during the two years shows that the runoff was concentrated from September to December. The total runoff at Wudongde was 937 × 10^8^ m^3^ from July to September, and it accounted for about 41.1% of the total annual runoff. The same amount was recorded at Baihetan. A comparison between the runoff from Baihetan and Wudongde shows that the amount from Wudongde was basically in the same order of magnitude as the outflow at Baihetan, which indicates that incoming water from the main stream is the main component of the inflow to Baihetan.

**Figure 5:**
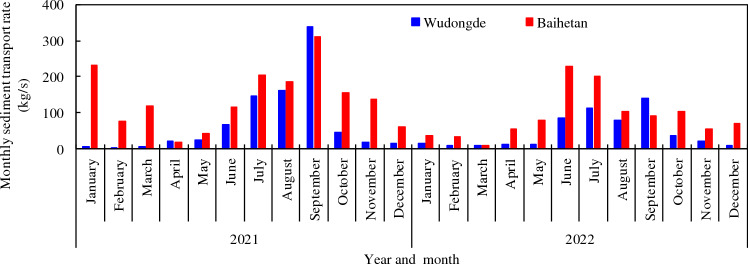
Monthly intake and output sediment transport rate from Baihetan Reservoir.

The comparison of the sediment transport rates for each month of the year (Fig. 5) shows that the amount of sediment transported from Baihetan was higher than that of Wudongde, except in September. This indicates that sediments from tributaries and the uncontrolled areas have a strong impact on Baihetan Reservoir.

#### Tributaries

**Figure 6:**
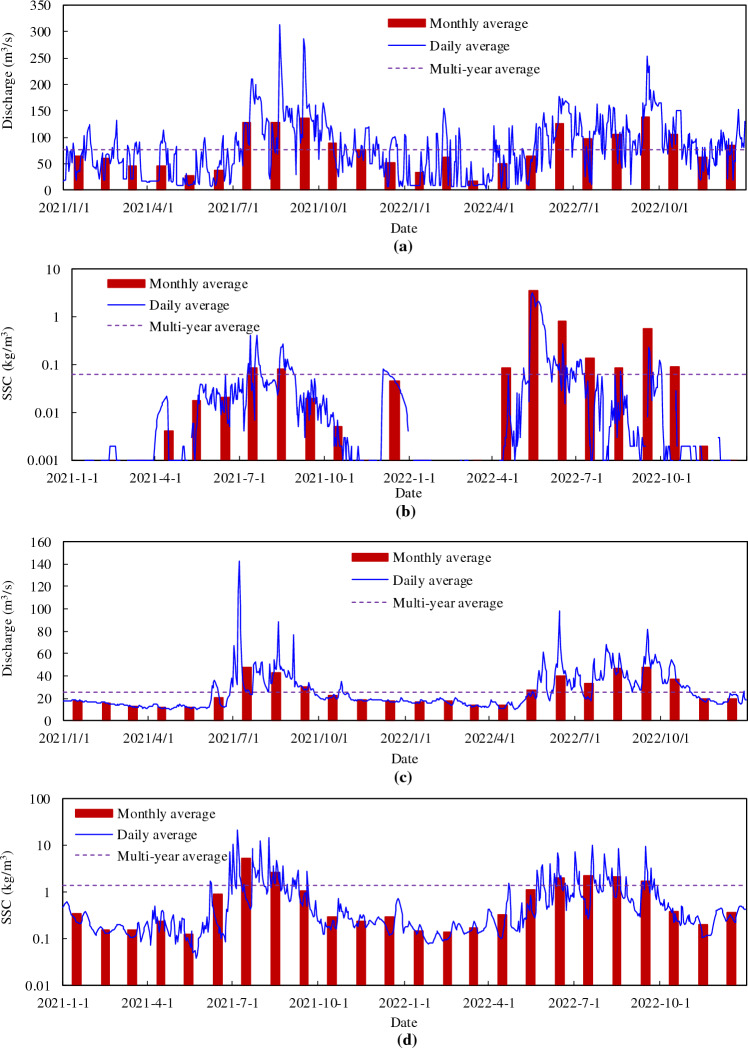
Main tributary flow and sediment processes: (**a**) flow process of the Pudu River; (**b**) sediment process of the Pudu River; (**c**) flow process of the Xiaojiang River; (**d**) sediment process of the Xiaojiang River.

**Figure 7:**
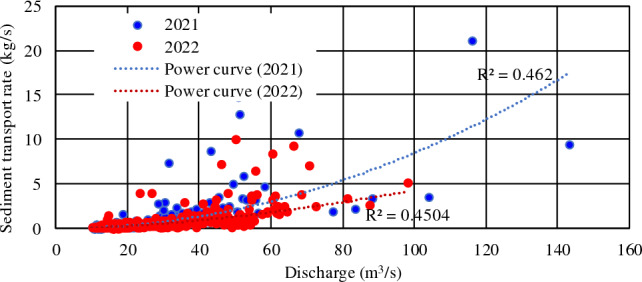
Correlation between daily average discharge and sediment transport rate at Xiaojiang station.

Table 4 shows the flow and sediment statistics of the main tributaries of Baihetan Reservoir, and Fig. 6 shows the corresponding daily average water and sediment processes. As the tributaries are mountainous rivers, characteristic sharp fluctuations are expected. The maximum annual flow discharges of the Pudu and Xiaojiang Rivers were 600 and 45 times the minimum discharges, respectively. The runoff and sediment transport processes were mainly concentrated from June to October. From 2021 to 2022, the total runoff from the Pudu and Xiaojiang Rivers was 47.04 × 10^8^ m^3^ and 15.989 × 10^8^ m^3^, accounting for 2.06% and 0.7% of the amounts recorded at Wudongde, respectively. The yearly sediment amount was 40.1 × 10^4^ t and 227 × 10^4^, accounting for 10.78% and 61.02% of the amounts recorded at Wudongde, respectively. The tributaries of the Baihetan Reservoir area have a relatively large amount of incoming sediment. In Xiaojiang, frequent floods and droughts occur in the watershed, and mudslides are the most severe. As many as 140 mudslides have been identified, and tens of valleys erode annually, making it a notorious mudslide area in China. Jiangjiagou Gully is the most representative debris-flow gully, and the Jiangjiagou mudslide blocked the Xiaojiang River seven times between 1919 and 1968. This attribute is evident in the relationship between the annual daily flow and SSC at Xiaojiang (Fig. 7). Although there is a certain correlation between the two; the greater the flow rate, the worse the correlation, indicating stronger randomness in sediment production. During 2022, the measured flow discharge increased from 2.45 to 112 m^3^/s, an increase of about 45 times, but the SSC amount varied from 0.078 to 45.1 kg/m^3^, an increase of about 577 times. Furthermore, the annual average SSC in 2022 was 93.5 times higher than that of Wudongde. The high SSC has had an obvious influence on deposition within the reservoir.

**Table 4:** Annual characteristic values of tributary flows and sediment in the Baihetan Reservoir area.

Tributary	Year	Runoff (10^8^ m^3^)	Sediment amount (10^4^ t)	Discharge (m^3^/s)	SSC (kg/m^3^)
Pudu river	2010–2021	22.0	37.2	69.8	0.169
	2021	22.22	18.4	70.3	0.083
	2022	24.82	21.7	78.7	0.087
Xiaojiang river	2020	8.773	139	27.8	1.58
	2021	7.191	119	22.8	1.65
	2022	8.798	108	27.9	1.22

Compared with the annual average, the annual runoff of the Pudu River from 2021 to 2022 was comparable with that from 2010 to 2021, but the sediment inflow amount was significantly lower. The amount of runoff and sediment from the Xiaojiang River were basically equivalent over the three years since its observation records began.

In summary, without considering sediment input from the uncontrolled sections, the total sediment input to Baihetan Reservoir from 2021 to 2022 was 639.1 × 10^4^ t, and the Pudu and Xiaojiang rivers accounted for 41.8% of this amount. However, this amount was lower than the approximate amount of 721 × 10^4^ t outgoing from Baihetan Reservoir. Therefore, sediment discharges from the uncontrolled section within the Baihetan area played an important role in the amount of sediment deposited within the reservoir area during the study period.

#### Sediment production in the uncontrolled area

Light and medium sediment yield areas are found above Panzhihua. Medium sediment yield areas mainly range from the Shigu to the Yalong River Estuary, and light sediment yield areas are mainly distributed above Shigu. The area below Panzhihua is characterized by high mountains and deep valleys, the downcutting of rivers, complex geological structures, developed faults, and strong gully erosion of most tributaries and creeks; frequent landslide and debris flows occur under gravity and hydraulic action. Affected by the geology, geomorphology, precipitation, and human activities, the LJR is the main source of the sediment into the Jinshajiang River, which is the key sediment-producing area. Soil erosion in this area is more serious than in any other area within the entire YRB, and it is the key soil and water conservation area.

According to a survey of remote sensing data in the 1990s, 400 landslides with a volume of more than 1 × 10^4^ m^3^ occurred within 15 km of both banks of the lower Jinshajiang River, which has an estimated volume of 3 × 10^8^ m^3^. In the LJR, there are a total of 438 general tributary gully debris flows with a drainage area greater than 0.2 km^2^, an accumulation fan area greater than 0.01 km^2^, 76 secondary or above tributary gully debris flows, and 37 main and tributary slope debris flows. In other words, there is one debris-flow gully every 1.8 km in the main Jinshajiang River, which is distributed between Panzhihua and Yibin. In addition, ancient forests in the area have been severely damaged, the slope farmland on both sides of the river valley is densely distributed, and heavy rainfall during the flood season causes serious water and soil loss.

According to statistics, the annual average suspended load sediment discharged at Baihetan station was 15,600 × 10^4^ t from 1958 to 2020, and the annual average sediment discharge modulus was 343 t/km^2^ a. The annual average suspended load sediment discharged at Xiangjiaba station from 1956 to 2020 was 20,600 × 10^4^ t, and the annual average sediment discharge modulus was 449 t/km^2^ a. The sediment transport modulus from Panzhihua to Baihetan was 1913 t/km^2^ a (excluding the Yalong River). With this sediment transport modulus, the annual sediment yield within 9059 km^2^ of uncontrollable areas, such as the Yili River and other unobserved tributaries in the Baihetan Reservoir area, was approximately 1733 × 10^4^ t, which was about 9.3 times that of the mainstream. With the addition of the sediment inflow from the Pudu River and Xiaojiang River, the total yearly sediment inflow from tributaries and uncontrollable areas was approximately10 times that of the mainstream.

### Sediment deposition

#### Mainstream

**Figure 8:**
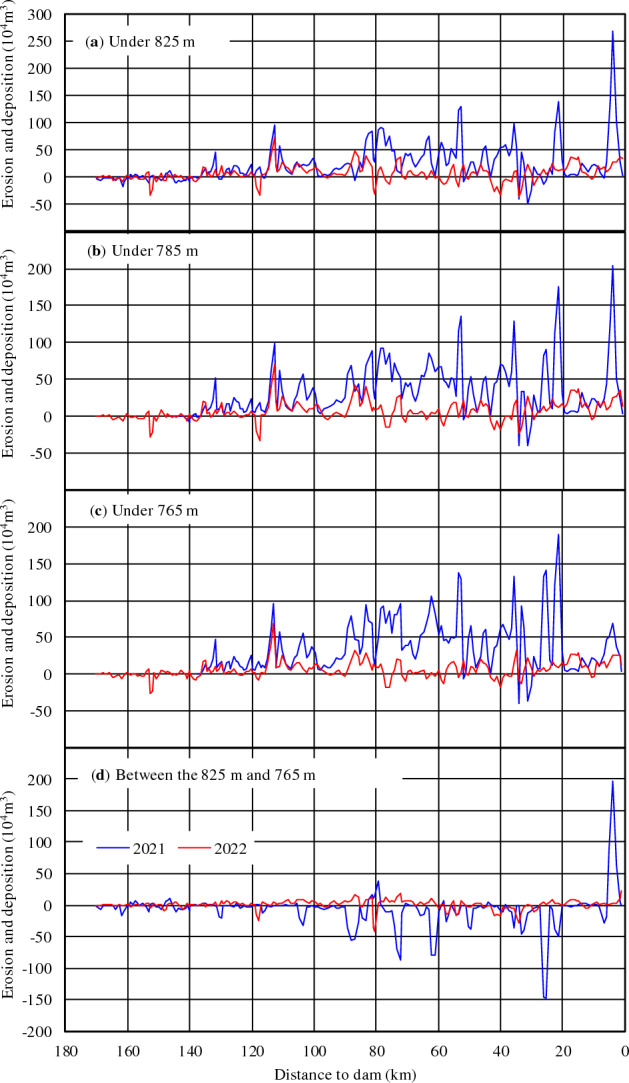
Distribution of sedimentation along the trunk stream of Baihetan Reservoir under different water levels for 2021 (blue) and 2022 (red).

Table 5 lists the amount of sediment deposited from the mainstream in the reservoir area; the amounts correspond to different water levels, and their distribution along the river is shown in Fig. 8. It is evident that from March 2021 to October 2022, an accumulated sediment amount of 5812 × 10^4^ m^3^ was deposited from the main stream to the reservoir area with an average sedimentation intensity of 34.3 × 10^4^ m^3^/km. The distribution characteristics along the river were as follows:

1. Variable backwater area (JC208–JC174): Influenced by the upstream Wudongde Reservoir, a minor amount of 94 × 10^4^ m^3^ was eroded from the tail section of the Baihetan Reservoir, with an erosion intensity of 3.95 × 10^4^ m^3^/km. Erosion mainly occurred in the river, approximately 10 km from the tail of the Wudongde Reservoir. At a distance of 160 km upstream of the Baihetan Reservoir site, the overall cross section was dominated by deposition.
2. Perennial backwater area (JC174–JC002): A siltation amount of approximately 5906 × 10^4^ m^3^ accumulated in this area, of which the downstream siltation intensity of the Xiaojiang confluence was higher than the upstream. The siltation intensity of the section from JC097 to JC066, adjacent to downstream of the Xiaojiang confluence, was the highest at approximately 61.2 × 10^4^ m^3^/km, which is close to 1.8 times the average intensity in the reservoir area. The second-highest intensity occurred in the reach from JC034 to JC002 near the dam, with a siltation intensity of 45.8 × 10^4^ m^3^/km.

**Table 5:** Scouring and silting amounts under different water levels in the trunk stream of the Baihetan Reservoir.

Reach	Length (km)	Amount of deposition (10^4^m^3^)								
		2021			2022			2021–2022		
		825 m	785 m	765 m	825 m	785 m	765 m	825 m	785 m	765 m
JC208–JC174	23.8	− 31	1.6	0	− 63	− 47	− 52	− 94	− 45.4	− 52
JC174–JC130	29.2	278	386	381	111	72	97	389	458	478
JC130–JC097	30.3	668	934	940	526	435	383	1194	1369	1323
JC097–JC066	26.9	1385	1739	1821	262	273	167	1647	2012	1988
JC066–JC034	27.5	1288	1462	1604	− 54	104	118	1234	1566	1722
JC034–JC002	31.5	926	1163	1038	516	530	422	1442	1693	1460
All	169.2	4515	5686	5785	1297	1366	1135	5812	7052	6920

The sedimentation characteristics at different elevations were as follows: sediment deposition in the trunk stream of the Baihetan Reservoir area were mainly accumulated within the dead storage capacity. An amount of 6920 × 10^4^ m^3^ was accumulated below 765 m, accounting for 0.81% of the dead storage capacity. The regulated storage capacity area between 765 and 825 m was in an undercutting state with an erosion amount of 1108 × 10^4^ m^3^, as shown in Fig. 8d. According to the statistics for the cross-sectional terrain, sections with significant erosion experienced landslides on the reservoir bank, with a total erosion amount of 297 × 10^4^ m^3^.

#### Tributaries

Statistics associated with riverbed erosion and deposition in the mouth section of the tributaries of the Baihetan Reservoir from 2021 to 2022 are listed in Table 6. From 2021, the Pudu, Daqiao, Xiaojiang, Yili, and Heishui River estuaries accumulated approximately 1840 × 10^4^ m^3^ of deposited sediments, of which the Xiaojiang River and Heishui River estuaries accumulated the largest amounts of 481.7 × 10^4^ m^3^ and 1176.9 × 10^4^ m^3^, respectively, accounting for 26.2% and 64.0% of the total sediments amounts deposited. The deposition intensity was 50.4 × 10^4^ m^3^/km and 45.9 × 10^4^ m^3^/km, respectively, which is close to the intensity of the mainstream in the reservoir area. Siltation amounts at the estuaries of the Pudu, Daqiao, and Yili rivers were lower and accounted for only 9.8% of the total amount of sediment deposited.

**Table 6:** Scouring and silting amounts under different water levels in tributaries within the Baihetan Reservoir area.

River	Length (km)	Amount of deposition (10^4^ t)								
		2021			2022			2021–2022		
		825 m	785 m	765 m	825 m	785 m	765 m	825 m	785 m	765 m
Pudu River	9.05	76.7	71.7	15.7	27.6	0.6	2.1	104.3	72.3	17.8
Daqiao River	2.38	3.2	− 1.7	− 1.4	8.7	10.4	18.9	11.9	8.7	17.5
Xiaojiang River	9.56	248.2	301.4	135.2	233.5	147.5	91.9	481.7	448.9	227.1
Yili River	7.24	83.3	116.6	117.5	− 18.7	− 7	4.7	64.6	109.6	122.2
Heishui River	25.63	504.5	433.8	398.5	672.4	537.8	437.7	1176.9	971.6	836.2
All	53.86	916	922	666	924	689	555	1840	1611	1221

During this period, 1221 × 10^4^ m^3^ of sediment was accumulated below the dead-water level (765 m), accounting for 0.14% of the dead storage capacity of the reservoir and 66.4% of the total amount deposited. A sediment deposition amount of 619 × 10^4^ m^3^ was accumulated in the regulated storage capacity between 765 and 825 m, accounting for 0.06% of the regulated storage capacity.

Overall, a total sedimentation volume of 8192 × 10^4^ m^3^ was accumulated below the dead-water level in the mainstream and tributaries from 2021 to 2022, was which was 15.8 times that of the regulated storage capacity (517 × 10^4^ m^3^). Therefore, the dead storage capacity areas of the mainstream and tributary estuaries in the perennial backwater area are the main siltation areas.

## Discussion

### Reservoir regulation

In the design phase of the Baihetan Reservoir, Lin et al.^43^ used a one-dimensional numerical model to predict sediment deposition in the reservoir. With the continuous promotion of cascade reservoir development, Huang et al.^36^ used a numerical model of the joint operation of cascade reservoirs to predict and calculate sediment deposition in Baihetan Reservoir. Although the flow and sediment boundaries used for predictions have varied in different periods, comparisons with the actual process helps to deepen our understanding of reservoir sediment deposition characteristics and guides optimization of the joint operation of the reservoir groups. Table 7 provides a comparison between predictions and the actual situation in Baihetan Reservoir recorded over the past 2 years. Compared to the results of this study, the two prediction results differ significantly from the actual measurements spanning the past 2 years, with respect to both the sediment conditions and the amount deposited. The input sediment used in the study by Lin et al.^43^ was approximately 3.4 times the actual value recorded in the 2 year period studied here, and their modelled sediment discharge ratio was approximately 43.07%, which was approximately 2.45 times the actual value. However, the predicted sediment-deposition intensity was essentially the same as the actual intensity. The input sediment used in the study by Huang et al.^36^ was approximately 2.72 times the actual value, with a sediment discharge ratio of approximately 13.71%, which was consistent with the actual value. However, the predicted deposition intensity was significantly greater than the measured value, at 1.56 times. In other words, both the sediment inflow used in the prediction but also the predicted sediment discharge ratio differed significantly from the actual values. This provides important insights for the optimization and operation of cascade reservoirs.

**Table 7:** First level tributary information from the Baihetan Reservoir area.

Achievements	Water and sediment series	Sediment entering the reservoir (10^8^ t/a)	Sediment discharge ratio (%)	Deposition intensity (10^8^ m^3^/a)
Reference^43^	1961–1970	0.698	43.07	0.331
Reference^36^	1991–2000	0.558	13.71	0.598
This study	2021–2022	0.205	17.56	0.383

The difference between the results of earlier studies and ours are related to two main causes: first, changes in system parameters. In this respect, when reservoirs were designed and modelled, there was an insufficient understanding of changes in sediment production under the influence of climate change and human activities. Cascade reservoirs have been built to store water in the middle and lower reaches of the Jinshajiang River since 2010, including in the main and tributaries of the Yalong River, and joint operations have gradually evolved. However, factors such as changes in basin rainfall and the application of soil and water conservation projects have significantly changed the water and sediment conditions in the lower reaches of the Jinshajiang River compared to those reported in the design and demonstration phase. Second, the amount of sediment input from the uncontrolled areas in the region are uncertain, and such amounts are severely influenced by climate and geology, which makes it difficult to accurately estimate sediment inflow conditions. Debris flow caused by local rainstorms and landslides caused by bank slope instability produce large amounts of sediment, which means that it is difficult to predict the amount of incoming sediment to the reservoir area. The lack of monitoring of these phenomena have resulted in an inability to provide accurate boundary conditions for reservoir designs and demonstrations.

It is necessary to obtain certain information to optimize the future design or cascade reservoirs and to predict the SSC and sedimentation. First, it is necessary to clearly understand climate change processes within the research area and to conduct trend analyses of long-term meteorological data. Second, the impact of human activities, such as hydropower development and the implementation of soil and water conservation projects, should be fully considered. Plans relating to human activities in the Jinshajiang River have been compiled, and these can be used as a reference. Adequate numbers of sediment production monitoring facilities are also required, and relevant parameters need to be calibrated based on specific circumstances, as certain theories describing sediment movement are not yet mature. At present, several reservoirs have been built in the Jinshajiang River and plenty of field data are available; such data are very useful for calibrating relevant parameters. Such insights will be helpful in the future analysis of the inflow water and sediment conditions and for verifying the effectiveness of model parameters.

### Reservoir sediment monitoring

According to the results of this study, the total annual amount of sediment input and output to and from the Baihetan Reservoir from 2021 to 2022 was 2052.6 × 10^4^ t and 360.5 × 10^4^ t, respectively. According to results based on the dry bulk density of sedimentation for the Xiluodu Reservoir downstream of Baihetan^46^, the amount from the main stream varied from 0.23 to 1.23 t/m^3^ (mean value 0.776 t/m^3^) and that of the tributaries ranged from 0.72 to 1.07 t/m^3^ (mean value 0.871 t/m^3^) from 2016 to 2019. Based on the average value of the main reservoir and tributaries, the annual volume of sediment deposited in Baihetan Reservoir over the two-year period was approximately 1918 × 10^4^ m^3^. According to the results of the terrain method used in this study, the annual sediment deposition volume over the 2 years was 3826 × 10^4^ m^3^, which was almost twice that determined using the sediment discharge method. The dry bulk density has an important impact on the analytical results associated with reservoir sediment deposition, and it should be an important focus of reservoir sediment monitoring.

**Figure 9:**
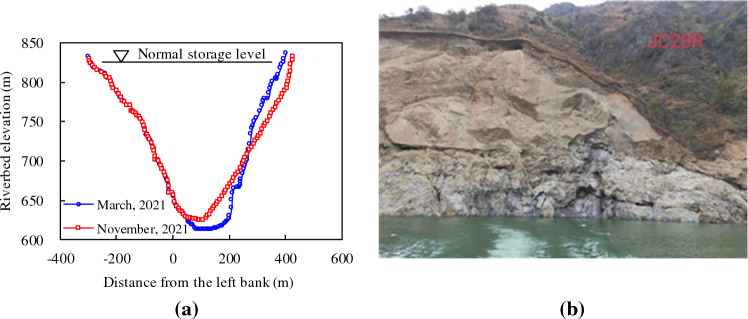
Typical landslide in the Baihetan Reservoir: (**a**) changes in cross-sectional morphology; (**b**) photo of landslide area.

The significant difference between the deposition amounts calculated using the sediment discharge method and those obtained using the terrain method may be due to the consideration of uncontrolled sediment inflow, such as that associated with mudslides and landslides in local areas. The main reach of the Baihetan Reservoir lies within an area that has the most concentrated numbers of landslides and debris flows in the entire LJR. After the reservoir is filled with water, erosion by the water body further affects the stability of mountains, causing sediment from landslides occurring on several mountains located above the normal water level (825 m) to enter the reservoir area, thereby leading to deposition in the reservoir area. For example, from March to November 2021, a local landslide occurred at an elevation of 710–850 m on the right bank (section JC028, 26.3 km upstream of the Baihetan Dam site). The sliding soil accumulated in the main channel, with a maximum accumulation amplitude of 35.3 m in the local area. As a result, the deep slope swayed towards the left bank with a swing amplitude of approximately 12.7 m, whereas the slope on the right bank slowed, as shown in Fig. 9. Therefore, after reservoir inception, geological hazard monitoring should be strengthened to further determine the amount of sediment input to the uncontrolled section of the reservoir.

## Conclusions

Incoming water, sediment and its deposition in the Baihetan Reservoir were studied using the latest hydrological and topographic data measured within the 2 years following the initial reservoir impoundment period. The results indicated that incoming water from the mainstream of the Jinshajiang River was mainly concentrated, and the maximum amount of water was stored in September. Discharge increased after the flood season. In the 2-year period, less sediment inflowed from the mainstream than the amount outflowing from Baihetan. Sediments from tributaries and uncontrolled sections of the reservoir area were important sources of sediment deposition in the reservoir.

During the 2 years studied, there was a slight erosion trend noted in the fluctuating backwater area of the reservoir area due to the influence of the upstream Wudongde Reservoir. In contrast, the perennial backwater area was in a clear siltation state, with 84% of the siltation occurring within the dead storage capacity. All estuaries of the tributaries in the reservoir area were in a siltation state, with 66.4% of the sediment deposited within the dead storage capacity, and this mainly occurred in the Xiaojiang and Heishui Rivers where the SSC was high.

Compared to the actual situation presented in this study, previous predictions have used greater sediment inflow boundaries and have predicted a greater siltation intensity. Therefore, further consideration should be given to the flow and sediment boundaries and the accuracy of mathematical models employed in future research that focuses on the optimal operation of cascade reservoirs. In addition, the difference between the amount of sediment deposited calculated based on the sediment transport rate and that based on the cross-section terrain determined in this study shows that it is necessary to further strengthen the measurements and analytical techniques used to determine the dry bulk density of reservoir sedimentation and any possible geological disasters that could occur in the reservoir area. This will help to gain a deeper understanding of the depositional law of the reservoir and provide support for the joint optimal regulation of cascade reservoirs.

## Data Availability

The datasets used during the current study are available from the corresponding author on reasonable request.
